# Revisiting renin-angiotensin-aldosterone system in aging: translational insights from bench to bedside and back

**DOI:** 10.1172/JCI195633

**Published:** 2025-11-03

**Authors:** Caglar Cosarderelioglu, Peter M. Abadir

**Affiliations:** Johns Hopkins University School of Medicine, Division of Geriatric Medicine and Gerontology, Baltimore, Maryland, USA

## Abstract

The renin-angiotensin-aldosterone system (RAAS) is a central regulator of cardiovascular, renal, and fluid homeostasis. Over the past century, our understanding of RAAS has evolved from a unidimensional circulatory hormone system to a complex network that includes local and intracellular signaling pathways. Aging profoundly impacts this system, influencing both systemic and tissue-specific RAAS activity. While levels of systemic RAAS components, such as plasma renin and aldosterone, decline with age, local RAAS components, particularly the proinflammatory angiotensin (Ang)II/AngII type 1 receptor (AT1R) axis, are upregulated in aging tissues, contributing to vasoconstriction, oxidative stress, inflammation, and fibrosis. Conversely, the protective arms of RAAS, the AngII/AT_2_R and Ang-(1–7)/Mas receptor pathways, are downregulated. Recent advances in geroscience have further illuminated how RAAS intersects with fundamental aging mechanisms, providing a mechanistic framework for understanding RAAS not only as a driver of age-related disease but also as a modifiable contributor to the aging process itself. In this Review, we summarize the evolution of RAAS biology, examine the molecular and functional consequences of aging on RAAS activity, and discuss the translational relevance of these findings. Finally, we explore emerging therapeutic strategies targeting RAAS components as potential interventions to promote healthy aging and reduce age-related disease burden, emphasizing a translational arc moving from bedside to bench and back, with the ultimate goal of improving patient outcomes.

Aging is accompanied by a broad spectrum of physiological changes that influence cardiovascular, renal, and metabolic function. Among the systems most sensitive to these changes is the renin-angiotensin-aldosterone system (RAAS). Alterations in RAAS with age are increasingly linked to age-associated disorders and geriatric syndromes. This Review examines age-related RAAS changes and their biological and clinical implications in the context of aging.

## Overview of the RAAS

**Figure 1:**
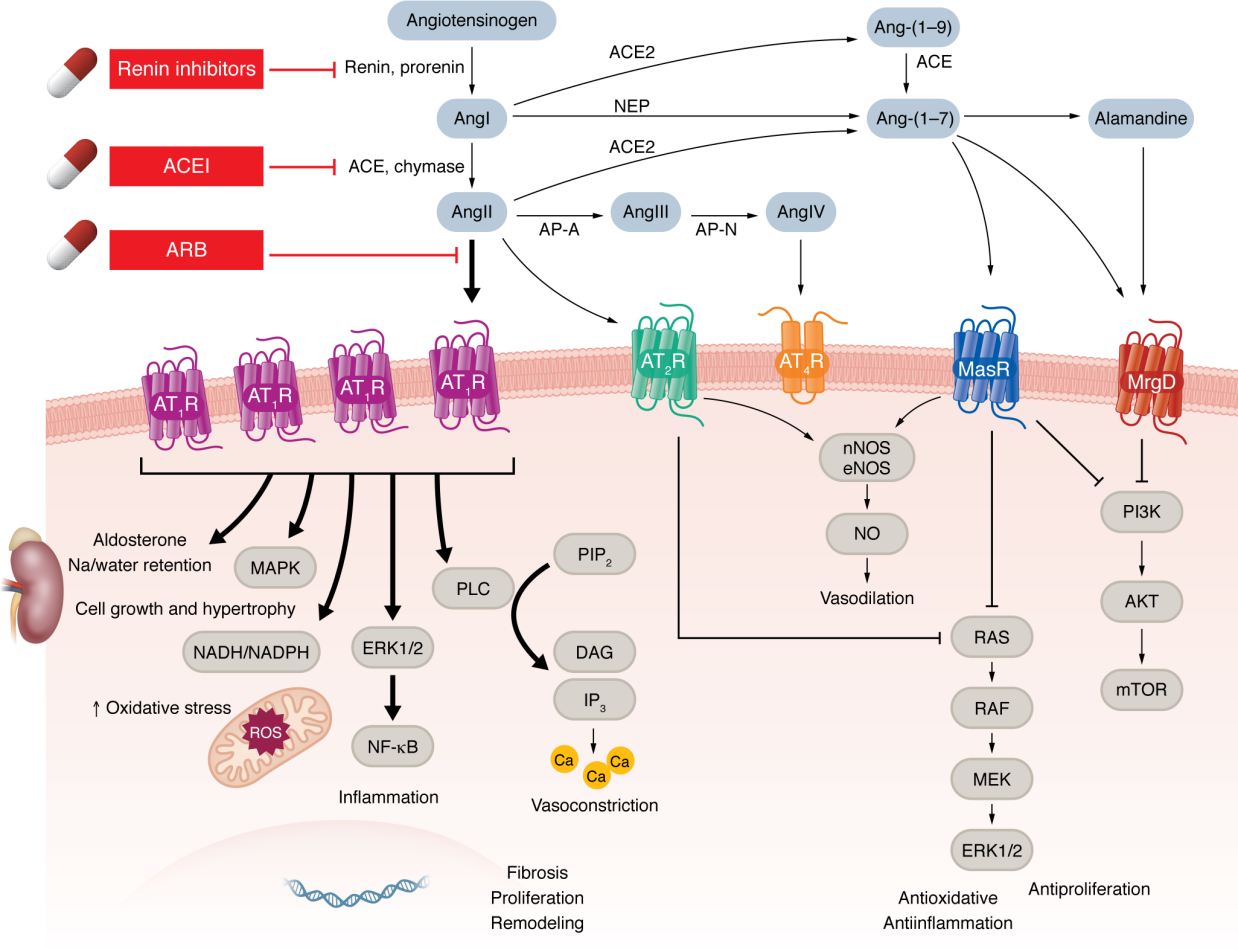
Summary of the renin-angiotensin-aldosterone system. As the first and rate-limiting step, renin converts angiotensinogen, a precursor molecule, into the decapeptide AngI. AngI is subsequently converted into AngII by ACE, a zinc metalloprotease predominantly found in the endothelial cells of the lungs (3). Cathepsin and chymase are also capable of hydrolyzing AngI to AngII. AngII exerts opposing effects: vasoconstriction through its binding to AT_1_R and vasodilation via AT_2_R. AngII promotes aldosterone production in the adrenal gland zona glomerulosa by enhancing the function of the steroidogenic acute regulatory (StAR) protein and aldosterone synthase, and causes an increase in sodium retention and potassium expulsion, resulting in a rise in water retention and BP. Glutamyl aminopeptidase A (AP-A) cleaves the N-terminal aspartate residue from AngII, producing the heptapeptide AngIII, which is subsequently converted to the hexapeptide AngIV by alanyl aminopeptidase N (AP-N) through cleavage of the N-terminal arginine. AngIV can then be further metabolized into Ang-(3–7) by the action of carboxypeptidase P and prolyl oligopeptidase. Alternatively, AngII can be converted into a heptapeptide Ang-(1–7) by carboxypeptidase P and ACE2, an isoform of ACE (3). ACE2 also catalyzes the conversion of AngI to Ang-(1–9), which can then be converted into Ang-(1–7) by ACE or produced directly from AngI via neutral endopeptidase. A newly identified component of the RAAS is alamandine, which is formed either by decarboxylation of Ang-(1–7) or through ACE2-mediated cleavage of angiotensin A—derived from the decarboxylation of AngII (5). ACE, angiotensin-converting enzyme; ACE2, angiotensin-converting enzyme 2; AngI, angiotensin I; AngII, angiotensin II; AngIII, angiotensin III; AngIV, angiotensin IV; AT_1_R, angiotensin II type 1 receptor; AT_2_R, angiotensin II type 2 receptor.

The RAAS was initially identified as a component of the endocrine system responsible for regulating water and electrolyte balance and systemic vascular resistance. The classical RAAS cascade (detailed in Figure 1) is initiated by release of renin into the bloodstream by the juxtaglomerular cells (JG cells) of the renal afferent arterioles, which store prorenin. Prorenin is cleaved into its active form, renin (1), and released into the bloodstream in response to specific physiological stimuli, including changes in renal BP, variations in sodium chloride concentration, increased sympathetic nervous activity, and feedback from humoral factors such as potassium (1, 2). In a rate-limiting step, renin converts the precursor angiotensinogen into angiotensin I (AngI), which is subsequently converted into AngII by angiotensin-converting enzyme (ACE) (3). AngII acts as the first major bioactive molecule, exerting opposing effects by binding to AngII receptors type 1 and 2 (AT_1_R and AT_2_R). Activation of AT_1_R promotes vasoconstriction, cellular proliferation, growth, and generation of ROS such as superoxide, whereas AT_2_R activation induces vasodilation and reduces inflammation, oxidative stress, and fibrosis (4). Details on receptors are provided in Table 1.

In addition to its systemic endocrine functions, the RAAS also operates via autocrine and paracrine mechanisms within specific tissues. While aldosterone is primarily synthesized in the adrenal cortex, several key components of the RAAS, such as angiotensinogen, renin, ACE, angiotensin receptors, and mineralocorticoid receptor, can be locally synthesized in tissues including the heart, kidneys, and brain (5, 6), referred to as the tissue RAAS. In the intracrine RAS (renin-angiotensin system), the system’s peptide effectors are synthesized and remain entirely within the originating cells. Notably, while angiotensin receptors are classically located on the plasma membrane, they have also been identified in high abundance within intracellular compartments, including the nucleus, mitochondria, and neurosecretory vesicles (7–12). Tissue RAS components can be regulated independently and play specialized roles in organ development, repair, fibrosis, inflammation, and remodeling. AngII, renin, and ACE can be localized to cytoplasm and nuclei, while ACE has been found on the endoplasmic reticulum (ER) (12, 13). Additionally, RAS components were detected in exosomes isolated from blood and urine of patients with hypertension (14).

## Impact of aging on the RAAS

Numerous studies have demonstrated that systemic RAAS activity diminishes with advancing age. Compared with younger individuals, older adults exhibit lower baseline plasma renin and aldosterone levels (15, 16). Additionally, the responsiveness of renin release to physiological stimuli, such as upright posture, sodium depletion, and hypotension, is markedly blunted in older adults (17, 18). Experimental models in aging animals have also confirmed decreases in both the synthesis and release of renal renin, contributing to the observed decline in circulating renin levels (19, 20). Several mechanisms have been proposed to explain this age-associated suppression of systemic RAAS. These include elevated arterial pressure leading to baroreceptor-mediated inhibition of renin release; reduced β_1_-adrenergic receptor sensitivity in renal tissues; and in some cases, impaired sympathetic innervation to the JG cells, particularly in individuals with autonomic dysfunction (21, 22). Despite declines in both renin and aldosterone levels with age, the aldosterone-to-renin ratio (ARR) tends to increase (23, 24), which may signal renin-independent aldosterone production. A recent study identified accumulation of aldosterone-producing cell clusters (APCCs) in the adrenal cortex of aging individuals, which may contribute to autonomous aldosterone secretion (25).

Moreover, serum ACE activity has been shown to decline in healthy older men (26). Interestingly, studies in aging animals have shown that renal AngII content increases, despite the overall reduction in circulating RAAS activity. In animal studies, AngII production and AT_1_R expression and sensitivity are upregulated in various tissues during aging (20, 27, 28). For example, AngII levels were increased in kidney as were ACE, and AT_1_R levels in hearts and vasculature of aged rats and primates, potentially contributing to structural and functional renal and cardiac remodeling, and suggesting an important role in senescence (28–31). On the other hand, AT_2_R, a member of RAAS’s protective arm, becomes less effective with age (32–34). Expression of AT_2_R declines with age in endothelial, neural, and renal tissues, potentially weakening the counterregulatory balance within the RAAS (20, 35, 36).

Furthermore, the protective Ang-(1–7)/Mas receptor axis is also downregulated with aging in mice, rats, and humans (21, 37, 38). Overall, current evidence suggests that while systemic RAAS activity is generally maintained or reduced with age, tissue RAS appears to be regulated differently. Moreover, biological sex influences the effect of aging on RAAS enzyme activity: older men showed significantly lower ACE activity than both older women and younger men (26).

## RAAS and geroscience

**Figure 2:**
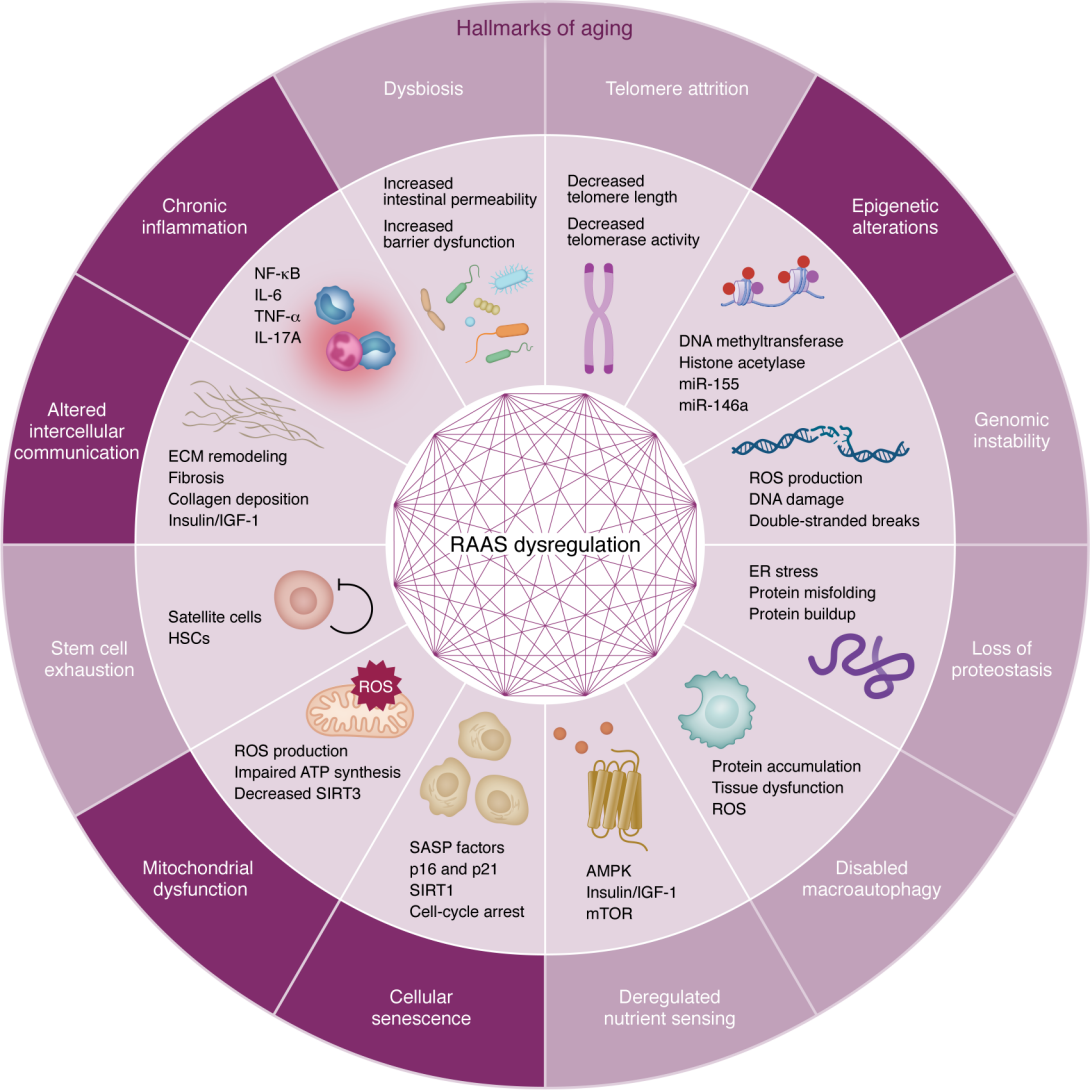
Dysregulated renin-angiotensin-aldosterone system and hallmarks of aging. Dysregulation of the renin–angiotensin–aldosterone system (RAAS) can contribute to each of the hallmarks of aging. AMPK, AMP-activated protein kinase; ATP, adenosine triphosphate; ECM, extracellular matrix; ER, endoplasmic reticulum; HSC, hematopoietic stem cell; IGF-1, insulin-like growthfactor 1; IL, interleukin; mTOR, mechanistic target of rapamycin; NF-κB, nuclear factor kappa-light-chain-enhancer of activated B cells; RAAS, renin-angiotensin-aldosterone system; ROS, reactive oxygen species; SASP, senescence-associated secretory phenotype; TNF-α, tumor necrosis factor-alpha.

Geroscience has emerged as a critical framework for investigating biological systems affected by aging, including the RAAS. Notably, RAAS signaling intersects with several of the hallmarks of aging, which were recently updated to include two new features: extracellular matrix (ECM) changes and psychosocial isolation (39, 40). This section explores how dysregulation of RAAS may contribute to the biology of aging in the context of these hallmarks (Figure 2).

### Epigenetic alterations.

The RAAS modulates epigenetic regulators such as DNA methyltransferases and histone acetyltransferases, leading to altered gene expression and cellular aging (41–44). Expression of RAAS-related genes is also regulated by epigenetic mechanisms, including DNA methylation, histone modifications, and miRNAs (45–47).

It has been shown that AngII modulates expression of histone demethylase 4A (KDM4A) and activation of class I HDAC1/2 (48). Consistent with this, silencing of HDAC1 or HDAC2 attenuated proliferation and migration of cardiac fibroblasts induced by AngII. Moreover, treatment with an HDAC inhibitor attenuated AngII-induced matrix metalloproteinase-9 and IL-18 expression and ECM production (collagen I, collagen III, and fibronectin) (49). Recent studies have revealed that, beyond stimulating expression of various genes linked to hypertrophy and fibrosis, AngII also influences cellular functions through epigenetic modifications in target cells (50). For example, AngII increases activity of class I HDAC1/2, leading to decreased histone acetylation at H3K9/14 and H4K8, which in turn suppresses Npr1 gene transcription. This downregulation reduces natriuretic peptide receptor-A protein expression and cGMP levels, ultimately impairing renal and vascular reactivity (51). Moreover, AngII promotes recruitment of SET1, a histone H3K4 trimethyltransferase, to the endothelin-1 promoter, and enhanced endothelin-1 expression contributes to persistent arterial hypertension and leads to organ damage, such as cardiac hypertrophy (52, 53).

### Loss of proteostasis.

Alterations in the RAAS can disrupt cellular protein homeostasis by increasing proteostatic stress and promoting the buildup of damaged or misfolded proteins via excessive activation of AngII signaling (4). This accumulation contributes to cellular dysfunction and accelerates the aging process. Research indicates that diseases associated with AngII share common age-related molecular disturbances such as impaired mitochondrial function and inadequate ER stress responses (54). These impairments foster formation of insoluble protein aggregates, which are worsened by reduced autophagic activity and the spread of cellular senescence (55). AngII contributes to ER stress through multiple pathways. Inhibiting the classical RAAS pathway can mitigate ER stress and inhibition of protein misfolding, while the counterregulatory arm of RAAS may offer protective effects (56, 57). Furthermore, disruptions in proteostasis can drive chronic inflammation by sustaining protein buildup, compounding the detrimental effects on cellular aging (54).

### Disabled macroautophagy.

The relationship between RAAS and macroautophagy is complex and context dependent. While AngII has been shown to induce autophagy in some cell types, including cardiomyocytes and neuronal cells, its effects vary with receptor subtype and physiological conditions (58). In cardiomyocytes, AT_1_R activation promotes autophagy, whereas AT_2_R appears to suppress it (59). Ang-(1–9)/AT_2_R was found to reduce both basal and AngII-induced autophagy in cardiomyocytes (60). Moreover, in neuronal PC12 cells, AngII triggers autophagy and apoptosis in an AT_1_R-dependent manner, reversible by the angiotensin receptor blocker (ARB) losartan (61). However, in renal proximal tubules, AngII or aldosterone infusion increases protein aggregates without upregulating autophagy, suggesting impaired proteostasis (54, 62). This aligns with the idea that in certain tissues, RAAS activation may exacerbate proteostatic stress without adequately triggering autophagic clearance. In hepatic stellate cells, AngII-induced ROS led to defective autophagosome clearance and fibrogenesis, and this was mitigated by alamandine, which shifted RAAS signaling toward the protective ACE2/MrgD axis (63). Low-dose AngII has also been shown to activate protective autophagy via ULK1 in renal tubular cells, suggesting a beneficial role in acute settings (64).

The dual nature of autophagy in response to AngII is further illustrated in cardiomyocytes under hypoxic conditions. Short-term AngII exposure may stimulate protective autophagy to mitigate cellular stress; however, with prolonged anoxia, apoptotic mechanisms become dominant. This temporal dynamic suggests that autophagy may serve as an early adaptive mechanism that becomes maladaptive if sustained (65).

### Mitochondrial dysfunction.

Age-related changes in the expression of mitochondrial RAAS receptors, with a shift toward increased AT_1_R over AT_2_R, stimulate NADPH oxidase and result in elevated mitochondrial ROS production. This elevation compromises mitochondrial integrity and function and alters mitochondrial membrane potential, reducing ATP synthesis and amplifying ROS and generation of peroxynitrite, a damaging molecule that disrupts the ETC (12, 66–68). Excess mitochondrial ROS are strongly linked to oxidation of mitochondrial proteins and lipids and to DNA mutations, which can drive cellular senescence and apoptosis (5, 11, 12, 67).

Supporting the detrimental impact of AT_1_R signaling is the finding that AT_1_R-knockout mice exhibit an extended lifespan and upregulation of mitochondrial and longevity-associated genes such as *Nampt* and *Sirt3* in the kidney (69, 70). Moreover, chronic activation of the AngII/AT_1_R/NOX pathway suppresses SIRT3 levels, increasing neuronal susceptibility to oxidative stress, and ARBs mitigate these detrimental effects in aged animal models (71). Intracellular AngII/AT_1_R/NOX signaling elevates superoxide levels, which uncouple eNOS, reducing NO bioavailability and mitochondrial NOS activity (72). Studies have also shown that ARBs and ACE inhibitors (ACEIs) are effective in reducing AngII-induced mitochondrial dysfunction and its associated deleterious effects (5, 73).

### Chronic inflammation.

Chronic, low-grade inflammation that increases with age — inflammaging — is a major contributor to the pathogenesis of age-related diseases and syndromes such as frailty (74). The RAAS plays a central role in driving this persistent inflammatory state. AngII promotes the production of proinflammatory cytokines such as IL-6, TNF-α, and IL-17A through activation of AT_1_R in macrophages and T lymphocytes (75–78). This cytokine release fuels systemic inflammation and contributes to endothelial dysfunction, arterial stiffness, and tissue fibrosis (4, 79). In addition, AngII upregulates adhesion molecules and chemokines, which enhance immune cell recruitment to vascular tissues, amplifying local inflammation and promoting atherosclerosis. Importantly, this is not a unidirectional pathway: inflammatory cytokines such as IL-6 can also stimulate expression of RAAS components such as angiotensinogen and renin, forming a self-sustaining proinflammatory loop (80–83). Furthermore, AngII exposure downregulates protective factors such as Klotho and PGC-1α, both of which decline with age and are essential for suppressing oxidative and inflammatory stress (75). Additionally, AngII modulates epigenetic regulators such as miR-155 and miR-146a that participate in the inflammatory aging phenotype (4, 84)​.

Counterbalancing this, components of the alternative RAAS axis — particularly through Mas receptor (MasR) and AT_2_R — exhibit antiinflammatory effects by inhibiting NF-κB signaling and cytokine release. Modulating RAAS signaling, either by inhibiting AT_1_R or enhancing protective pathways such as ACE2/Ang-(1–7)/Mas, offers a promising approach to mitigate inflammaging and the associated functional decline in multiple tissues.

### Cellular senescence.

Accumulating evidence indicates that the RAAS plays a role in the induction and progression of senescence, especially in vascular and renal tissues. Chronic, low-dose AngII exposure has been shown to promote senescence in kidney tissues and contribute to the formation of an inflammatory microenvironment through release of senescence-associated secretory phenotype (SASP) factors and recruitment of immune cells (85). Endothelial cells, in particular, appear to be highly susceptible to AngII-induced senescence. In the INK-ATTAC transgenic mouse model, elimination of senescent cells prevented AngII-induced inflammation and tissue damage, highlighting the therapeutic potential of senolytic interventions targeting RAAS-mediated senescence (85).

In vitro studies further demonstrate a biphasic response to AngII (86). While transient AngII exposure enhances endothelial functions such as proliferation, migration, and angiogenesis, prolonged exposure impairs endothelial viability; induces apoptosis; and upregulates senescence markers including senescence-associated β-gal (SA–β-gal) activity, p21, and proinflammatory cytokines. ARBs mitigated these effects in cell culture, suggesting a direct role for the AngII/AT_1_R axis in endothelial aging and dysfunction (86).

AngII also accelerates senescence in vascular smooth muscle cells (VSMCs), contributing to atherosclerosis development through a p21-dependent mechanism (87). This process involves AT_1_R-mediated activation of signaling molecules including Ras, MAPKs (such as ERK1/2), and transcription factors like NF-κB and AP-1, leading to oxidative stress and upregulation of cell-cycle inhibitors such as p53 and p21 (88). Notably, these detrimental effects are reversed by the ARB losartan, indicating that RAAS inhibitors (RAASi) may attenuate aging-associated cellular decline (89, 90).

Moreover, in mice, inactivation of AT_1_R has been associated with a reduction in AngII-induced cellular senescence markers. Mice lacking AT_1_R exhibited extended lifespan compared with controls, potentially due to reduced oxidative stress and enhanced expression of prosurvival genes. These findings suggest that AT_1_R plays a pivotal role in regulating senescence and aging at the cellular level (69, 91).

### Altered intercellular communication and ECM changes.

Progressive disruption of intercellular communication leads to systemic dysfunction and loss of homeostasis (39). The RAAS plays a role in this process, acting as a neurohormonal axis that not only regulates cardiovascular and renal physiology but also may contribute to age-related impairments in cellular signaling. With age, the AngII/AT_1_R axis becomes overactivated, which exerts proinflammatory, profibrotic, and pro-oxidative effects across multiple tissues, as mentioned above (92). AngII can induce SASP, amplifying local and systemic inflammatory signals that disturb paracrine and endocrine communication (85). Moreover, RAAS interacts with other aging-relevant signaling networks, including insulin/IGF-1, adrenergic, and dopaminergic pathways, contributing to the breakdown of hormetic regulation and adaptive stress responses (93).

Furthermore, RAAS activation contributes to ECM remodeling and fibrosis by promoting collagen deposition and altering matrix turnover (94). These structural changes compromise tissue integrity and elasticity, further accelerating age-related functional decline.

### Dysbiosis.

Bidirectional interplay between gut microbiota and the RAAS may influence age-related physiological decline and pathology (95). Dysbiosis becomes more prevalent with aging and is increasingly recognized as a contributor to inflammaging, metabolic disorders, and neurodegeneration (96). Under physiological conditions, local gastrointestinal (GI) RAS helps maintain homeostasis in digestion, electrolyte transport, immune modulation, and mucosal protection (95). Studies demonstrate that dysbiosis can alter local RAS activity. Some studies also suggest that microbiota-derived short-chain fatty acids (SCFAs) may modulate local RAS components through SCFA receptors expressed in the renal vasculature (97), indicating a potential bidirectional relationship between gut microbes and the RAS. In contrast, microbial modulation — through fecal microbiota transplantation or prebiotic/probiotic supplementation — can shift RAS activity toward the ACE2/Mas axis, attenuating age-related tissue damage and systemic inflammation, and improving outcomes in animal models of neurological aging (95, 96). The AngII/AT_1_R axis promotes inflammation and oxidative stress in the gut epithelium, contributing to increased intestinal permeability and barrier dysfunction (98). Conversely, counteracting the effects of AngII has been shown to partially restore microbial diversity, reduce intestinal inflammation, and improve epithelial barrier function (99, 100).

The relationship between additional hallmarks of aging and RAAS is summarized in Table 2.

## RAAS and lifespan

Both pharmacological interventions and genetic studies have highlighted the influence of RAAS on longevity. Inhibition of ACE homologs in *C*. *elegans* and *Drosophila* and long-term inhibition of AngII signaling in rodents have been associated with lifespan extension (101). For instance, chronic administration of ACEIs or ARBs has been shown to double the lifespan of hypertensive rats (102, 103). Similarly, treatment with the ACEI enalapril in normotensive Wistar rats not only reduced body weight gain but also extended lifespan, suggesting benefits beyond BP control (104). However, these studies did not directly compare RAASi with other antihypertensive drug classes such as calcium channel blockers, which limits conclusions regarding the specificity of RAAS blockade for lifespan extension.

Genetic studies further corroborate these findings. Mice lacking the AT_1A_ receptor (*Agtr1a^−/−^* mice) exhibit a 26% increase in lifespan compared with WT counterparts (69). This longevity is accompanied by reduced oxidative stress, diminished organ damage, and upregulation of prosurvival genes, such as *Sirt3*, *Nampt*, and *Klotho* (69).

In humans, polymorphisms in the *AGTR1* gene, which encodes AT_1_R, have been linked to exceptional longevity (105). Notably, the GG genotype of the rs275653 promoter variant was found to be more prevalent among centenarians and is associated with lower AT_1_R expression and reduced BP (105). These genetic associations suggest that diminished AT_1_R activity may confer protective effects against age-related pathologies (54).

Furthermore, AngII has been shown to suppress the expression of SIRT1 (106), a key regulator of cellular stress responses and longevity. This suppression exacerbates oxidative stress and promotes cellular senescence. Importantly, elevated circulating levels of AngII have been correlated with higher long-term all-cause mortality, independent of conventional cardiovascular risk factors (107).

Collectively, these findings underscore the pivotal role of the RAAS, particularly the AngII/AT_1_R axis, in modulating aging and lifespan. Therapeutic strategies targeting this pathway, whether through pharmacological agents such as ACEIs and ARBs or genetic modulation, hold promise for promoting healthy aging and extending lifespan (108).

## Geriatric syndromes, aging-associated diseases, and RAAS

Excessive activation of the classical RAAS pathway contributes to dysfunction in key organs such as brain, kidneys, vasculature, and skeletal muscle, ultimately promoting geriatric syndromes and conditions such as stroke, HF, and chronic kidney disease (CKD).

### Dementia, delirium, and depression.

The RAS is increasingly recognized as a player in the pathophysiology of neuropsychiatric disorders, including dementia, delirium, and depression. The AngII/AT_1_R axis contributes to neurodegeneration and dementia, including Alzheimer’s disease (AD), by promoting chronic inflammation, microglial activation, and oxidative damage — factors that accelerate neuronal injury and synaptic loss (109–111). Elevated AngII levels have been associated with reduced gray matter and hippocampal volume, both critical to memory function (112). Additionally, AngII is reported to increase brain amyloid-β levels via multiple mechanisms, such as increasing amyloid precursor protein mRNA, γ-secretase activity, and presenilin expression (113, 114). The brain RAS’s protective arm becomes less effective with aging due to a decline in AT_2_R expression (32–34). In aged animal models, this reduction in AT_2_R was associated with enhanced susceptibility to the effects of AT_1_R overactivation, including increased oxidative damage, inflammation, and neuronal vulnerability (36). Moreover, AT_2_R oligomerization triggered by amyloid-β may enhance neurodegeneration (115). In AD patient brain tissue, ACE has been shown to be elevated in the hippocampus, frontal cortex, and caudate nucleus regardless of hypertension, and the levels correlate with AD pathology (116, 117). Similarly, cerebrospinal fluid (CSF) ACE activity was found to be elevated in AD (118).

The AngII/AT_1_R axis can also contribute to AD via vascular changes such as constriction of cerebral vessels, vascular remodeling, impaired cerebrovascular autoregulation, and endothelial dysfunction (119, 120). In line with this, it has been shown that RAS overactivity is correlated with CSF markers of capillary damage such as elevated CSF-soluble PDGFRβ, indicating pericyte damage, and elevated CSF albumin, indicating blood-brain barrier (BBB) breakdown in AD (118). AngII/AT_1_R signaling can damage the BBB, increase its permeability, and reduce cerebral blood flow via its proinflammatory and pro-oxidant effects (121). Blockade of AT_1_R and activation of AT_2_R reverse hypertension-induced cerebrovascular dysfunction and improve barrier function of endothelial cells and diabetes-associated cerebral endothelial dysfunction (122–124).

AngII has been implicated in disruption of insulin signaling in the brain (125), indicating that RAS activation may also contribute to cognitive decline by altering insulin sensitivity. Finally, the cholinergic system — critical for learning and memory — is negatively influenced by RAAS overactivation (126, 127). AngII reduces acetylcholine release and impairs long-term potentiation (LTP), while activation of the alternative RAS axis, such as Ang-(1–7)/MasR, AngIV /AT4R, and the AngII/AT2R has been shown to enhance memory, synaptic plasticity, and neuroprotection (5, 128–131).

The observation that drugs targeting the RAAS, whether ACEIs, ARBs, or AT_2_R agonists, preserve memory and attenuate amyloid- and tau-related pathology in animal models, coupled with epidemiological and clinical findings linking RAASi use to a lower incidence and slower progression of AD in humans, provides further evidence that dysregulated angiotensin signaling actively contributes to AD (73, 108, 132–136).

Delirium may also be influenced by RAS activity. Elevated levels of AngII may impair cholinergic signaling and contribute to BBB dysfunction — both of which are factors implicated in delirium pathogenesis (109). Some observational studies suggest a lower incidence of delirium in patients treated with ARBs, though more research is needed (137, 138).

The RAS may influence mood regulation through several mechanisms, such as its effects on neurogenesis and hypothalamic-pituitary-adrenal axis activity (139–141). Increased AngII/AT_1_R signaling has been linked to elevated cortisol and depressive symptoms. Animal studies and limited clinical data suggest that RAASi may exert antidepressant-like effects, potentially enhancing serotonin and glutathione availability and reducing neuronal damage by decreasing oxidative stress, microglial activation, and levels of inflammatory markers such as TNF-α (141). Overall, RAAS modulation may offer neuroprotective and mood-stabilizing benefits, especially in older adults at risk (136).

### Frailty, sarcopenia, and falls.

Frailty and sarcopenia are highly prevalent geriatric syndromes characterized by a loss of physiological reserve and increased vulnerability to stressors and by a progressive decline in skeletal muscle mass, strength, and function, respectively (142, 143). These conditions substantially increase the risk of falls, hospitalization, dependency, and mortality in older adults (142–147).

Emerging evidence suggests a mechanistic link between the RAAS and the development and progression of both sarcopenia and frailty, mediated by inflammatory, oxidative, and mitochondrial pathways (20, 148). In animal studies, AngII associated with pronounced skeletal muscle atrophy, characterized by upregulation of the E3 ubiquitin ligases atrogin-1 and MuRF-1, increased proteolysis through the ubiquitin–proteasome system, and elevated NADPH and mitochondria-derived ROS generation (149, 150). Moreover, AngII decreased the number and size of regenerating myofibers and inhibited satellite cell regenerative capacity and muscle regeneration. Similar to AngII, agonistic AT_1_R autoantibodies are capable of activating the AT_1_R, and elevated concentrations of these autoantibodies have been associated with increased levels of inflammatory cytokines, reduced grip strength, slower gait speed, and a heightened risk of frailty and falls (151, 152).

In contrast to the catabolic influence of AngII/AT_1_R, components of the protective RAS arm protect muscle from pathological remodeling and muscle insulin resistance (150). For instance, ACE2-deficient mice exhibited premature muscle weakness, along with indicators of the aging process such as induction of p16INK4a, a senescence-associated gene, and aging-associated changes of myofiber structure, effects that were reversible upon Ang-(1–7) application (153).

Human studies increasingly support the clinical relevance of these mechanisms. The Singapore Longitudinal Ageing Study found that ARB use was associated with a reduction in frailty and age-related loss of muscle mass and strength (154), whereas ACEI use had less-consistent effects​ in different studies (154, 155). Further studies are needed to clarify the benefits of RAASi in maintaining functional independence.

### Pressure ulcers, impaired wound healing, and tissue regeneration.

The skin expresses multiple components of the RAAS within the epidermis, dermis, and hair follicles, and RAAS has role in key processes essential for wound healing, including cell migration, proliferation, collagen turnover, inflammatory response, and TGF-β signaling, as well as regulation of stem cell proliferation and differentiation, inflammatory responses, fibrosis and scarring, vascular tone, and even skin tumorigenesis (94, 156, 157).

During the early phase of wound healing, increased vascular permeability facilitates leukocyte infiltration and inflammation. Concurrently, epithelial stem cells and dermal fibroblasts are activated. ACE plays a critical role by converting AngI to AngII, thereby promoting stem cell migration and initiating tissue regeneration. In the later regenerative phase, AngII interacts with AT_2_R to activate the ERK and STAT1/3 pathways, which enhance fibroblast proliferation and granulation tissue formation. Additionally, RAS signaling stimulates the production of profibrotic molecules, supporting collagen deposition and matrix remodeling during the healing process (94).

Dysregulation of the skin RAS in aging, with increased AT_1_R and decreased AT_2_R expression, is implicated in abnormal wound healing (157–159). An imbalance in dermal expression of AT_1_R and AT_2_R has been linked to structural deterioration, including epidermal thinning, collagen degradation, disruption of the dermal architecture, and loss of subcutaneous fat in diabetic rats (159).

While excessive AT_1_R signaling impairs tissue repair by promoting fibrosis and delaying re-epithelialization, AT_2_R activation appears to facilitate regenerative processes (160). Experimental models demonstrate that topical RAAS modulation — particularly with ARBs such as valsartan — enhances healing outcomes by increasing wound blood flow, collagen deposition, and tissue tensile strength and enhancing re-epithelialization, neovascularization, and formation of organized granulation tissue (157, 161–163). These findings underscore the potential for tissue-targeted RAAS therapies to restore regenerative capacity in compromised healing environments.

### Polypharmacy.

Polypharmacy, often defined as the use of five or more medications, is common in older adults with multiple chronic conditions and increases the risk of adverse drug events. It is important to note that most research studies are conducted using a single medication, whereas in real-life settings, older adults typically take multiple medications. Medications that inhibit the RAAS are frequently prescribed to manage hypertension, HF, and CKD. However, significant interactions can occur when RAASi are combined with other drugs such as diuretics and NSAIDs, such as increased risk of acute kidney injury (AKI) due to synergistic effects on renal perfusion and function. A study found that combined use of an ACEI or ARB, a diuretic, and an NSAID raised the risk of AKI by 31% (164). Additionally, while each class offers therapeutic benefits, combined use of multiple RAAS-modifying drugs — referred to as dual or triple RAAS blockade — may be associated with an increased risk of adverse effects, and combination of these drugs must be considered more cautiously in older adults (165, 166). Careful medication review, monitoring, and individualized treatment based on risk are essential to minimize adverse outcomes in older adults.

### Urinary incontinence.

AngII inhibition has been shown to decrease both detrusor overactivity and urethral sphincter tone in animal models, leading to reduced urgency urinary incontinence (UUI) and increased stress urinary incontinence (SUI) (167). Similarly, in a population-based study (National Health and Nutrition Examination Survey [NHANES], 2001–2008), the use of ACEIs or ARBs was associated with a 25%–30% reduction in UUI in men. However, ACEI or ARB use was not linked with any changes in SUI in either men or women. No similar UUI reduction was seen with other antihypertensive drug classes, including diuretics, betablockers, or calcium channel blockers (167).

### Sleep disturbances.

Nocturnal average renin levels are lower in women than in men and decline with age, though their relationship with sleep appears independent of age and sex (168). Besides nocturnal regulation of renin, poor sleep quality has been linked to elevated plasma aldosterone levels, especially in young and middle-aged men and older women. Aldosterone levels increased progressively with worsening sleep quality, suggesting a potential role of sleep in modulating RAAS activity (169).

### Sensory impairments.

Recent research suggests that the RAAS may also influence sensory function in aging (170–173). Activation of AT_1_R and the renin/prorenin receptor (PRR) has been associated with the development of age-related macular degeneration (AMD). Preclinical studies indicate that treatments such as ARBs and ACEIs may help reduce choroidal neovascularization in AMD by suppressing inflammation (174, 175). Higher serum aldosterone levels have been associated with better hearing in older adults, and aldosterone may influence age-related hearing processes by upregulating BCL-2 expression and inhibiting pathways involving BAX and caspases (171, 173).

### Stroke.

The RAAS plays a central role in the pathophysiology of stroke through both systemic and tissue-specific mechanisms. In addition to its well-established contribution to hypertension, overactivation of the AngII/AT_1_R axis is one of the most significant modifiable risk factors for stroke, contributing to vasoconstriction, increased sympathetic tone, oxidative stress, inflammation, disruption of the BBB, and endothelial dysfunction, key processes in stroke pathogenesis (176). In the setting of cerebral ischemia, AngII/AT_1_R activation impairs perfusion to the penumbra, potentially exacerbating tissue damage (177).

In contrast, the alternative RAAS axis counteracts the effects of AngII/AT_1_R activation, exerting vasodilatory, antiinflammatory, and neuroprotective actions (5). Blockade of the AngII/AT_1_R axis may therefore confer benefits in two primary domains: the cerebral parenchyma, by modulating inflammation and promoting neuronal survival, and the cerebral vasculature, by restoring perfusion and preserving endothelial function (178). Both preclinical and clinical studies support the neuroprotective potential of RAASi through decreasing activity of AT_1_R. In rodent models of ischemic stroke, ARBs have been shown to reduce infarct size and improve neurological outcomes (179)​. In the Heart Outcomes Prevention Evaluation (HOPE) trial, the ACEI ramipril significantly reduced stroke risk, with effects likely attributable to RAAS modulation beyond BP control. The Microalbuminuria, Cardiovascular and Renal Outcomes–HOPE (MICRO-HOPE) substudy corroborated these findings, demonstrating greater cardiovascular protection than expected from BP reduction alone (180). Similarly, the Losartan Intervention for Endpoint Reduction (LIFE) trial reported that losartan was more effective than atenolol in stroke prevention, despite comparable antihypertensive efficacy (181). Observational data further suggest that RAASi are associated with reduced in-hospital mortality among patients with acute ischemic stroke (182). However, a large meta-analysis of 147 randomized trials by Wald and Law indicated that most of the stroke risk reduction associated with ACEIs and ARBs may be explained by their BP-lowering effects rather than BP-independent mechanisms (183).

Recent attention has turned to the RAAS-protective arm. Emerging therapeutic strategies targeting these alternative axes — such as AT_2_R agonists (e.g., C21, CGP42112), Ang-(1–7), and β-arrestin-biased AT_1_R agonists — are under active investigation for their potential to provide neuroprotection without the deleterious effects associated with classical AT_1_R stimulation (178).

### Atrial fibrillation and heart failure.

With advancing age, the dysregulated RAAS contributes substantially to the development and progression of atrial fibrillation (AF) and heart failure (HF), two highly prevalent and interlinked conditions in older adults. Overactivation of the AngII/AT_1_R axis promotes adverse structural, electrical, and neurohormonal remodeling through inflammation, oxidative stress, and fibrosis and accelerates cardiovascular aging via mechanisms such as mTOR activation and suppression of aging-protective molecules such as sirtuins (e.g., SIRT1), Klotho, and PGC-1α (54, 184). The RAAS stimulates fibroblast proliferation and ECM deposition via upregulation of MAPK signaling and reduced collagenase activity, contributing to atrial structural remodeling and electrophysiological disturbances that promote reentry circuits, involving a reduction in the atrial effective refractory period and shortening of action potential duration (185). Aldosterone further exacerbates this process by activating mineralocorticoid receptors, leading to left atrial dilation and fibrosis, particularly in patients with primary aldosteronism​ (186).

Similarly, in HF, persistent RAAS activation drives maladaptive remodeling. The AngII/AT_1_R axis promotes cardiomyocyte hypertrophy, apoptosis, interstitial fibrosis, vascular inflammation, and myocardial stiffening (187–189). AngII has also been shown to increase exosome release from cardiac fibroblasts, and these exosomes upregulate renin, angiotensinogen, AT_1_R, and AT_2_R, while downregulating ACE2 and enhancing AngII production, ultimately promoting hypertrophy (190). These changes contribute to both systolic and diastolic dysfunction and increase susceptibility to arrhythmias.

AF and HF frequently coexist and exacerbate one another in older adults, with the bidirectional relationship intensified by RAAS-mediated remodeling. HF increases left atrial pressure and volume, promoting atrial stretch and fibrosis, while AF contributes to reduced cardiac output and tachycardia-induced cardiomyopathy (191, 192). RAAS inhibition represents a key therapeutic strategy to break this pathological loop.

Treatment with ACEI and ARBs has been shown to attenuate cardiac remodeling by limiting left atrial enlargement, dysfunction, and fibrosis and shortening the atrial effective refractory period — changes that are expected to lower the risk of AF in animal models of HF (193). Modulation of RAAS with ACEIs, ARBs, and mineralocorticoid receptor antagonists (MRAs) was shown to reduce the incidence of new-onset and recurrent AF, especially in patients with hypertension or left ventricular dysfunction (194, 195). Importantly, the benefits of RAAS inhibition extend beyond hemodynamic control (196). In both AF and HF, ACEIs and ARBs exert antiinflammatory, antifibrotic, and antioxidative effects. Growing understanding of tissue-specific RAAS adds nuance, particularly in aging tissues where the protective ACE2/Ang-(1–7)/Mas and AT_2_R axes are suppressed​. The balance between the deleterious AngII/AT_1_R axis and the protective ACE2/Ang-(1–7)/MasR and AT_2_R pathways is crucial in determining the outcome in AF and HF. Therapeutic strategies aimed at enhancing the protective arm are currently under investigation and may represent the next frontier in RAAS-targeted therapy (197, 198).

Moreover, the Randomized Aldactone Evaluation Study (RALES), Eplerenone Post-AMI Heart Failure Efficacy and Survival Study (EPHESUS), and Eplerenone in Mild Patients Hospitalization and Survival Study in Heart Failure (EMPHASIS-HF) trials established that steroidal MRAs such as spironolactone and eplerenone significantly reduce mortality and hospitalizations in patients with HF with reduced ejection fraction (HFrEF), leading to their strong endorsement in international guidelines (199–201). However, spironolactone did not improve the primary composite outcome of HF hospitalization, resuscitated cardiac arrest, or cardiovascular death in patients with preserved ejection fraction (HFpEF) in the Treatment of Preserved Cardiac Function Heart Failure with an Aldosterone Antagonist (TOPCAT) trial (202). More recently, the Finerenone Trial to Investigate Efficacy and Safety Superior to Placebo in Patients With Heart Failure (FINEARTS-HF) trial and a meta-analysis by Jhund et al. showed that nonsteroidal MRAs led to cardiovascular benefits in patients with mildly reduced ejection fraction (HFmrEF) or HFpEF (203, 204).

### CKD.

With advancing age, both circulating and intrarenal components of the RAAS undergo marked shifts that predispose the aged kidney to functional decline and structural injury. Plasma renin and aldosterone levels fall, whether at baseline or in response to classical stimuli, and older individuals consequently lose much of their capacity for sodium conservation and potassium excretion, placing them at risk for salt wasting hyponatremia and hyperkalemia (205).

Healthy kidneys predominantly convert AngI to the vasodilator Ang-(1–7) via neprilysin, with a fraction shunted to AngII by ACE. In CKD, this balance reverses: Ang-(1–7) formation falls, while AngII rises, partly because local generation of AngII shifts from ACE toward chymase, an enzyme unleashed by tissue injury (206, 207).

These RAAS alterations have profound hemodynamic and structural consequences. Aged kidneys commonly exhibit afferent arteriolar hyalinization, exaggerated glomerular hypertension, and accelerating sclerotic changes (16, 205). In animal models, mesangial expansion, interstitial fibrosis, and proteinuria can be ameliorated by ACEIs or ARBs (16, 208, 209). In patients with mild or moderate CKD, ARBs and ACEIs reduce BP, slow estimated glomerular filtration rate (eGFR) decline, reduce proteinuria, and delay progression to advanced CKD; however, the effects of ARBs and ACEIs on mortality and cardiovascular risk in patients with CKD remain controversial (210, 211). A recent study showed that aging does not modify RAASi’s beneficial effects on CKD outcomes or their potential adverse effects (211).

### Therapeutic modulation of RAAS in aging.

As discussed above, the RAAS is increasingly recognized as an important therapeutic target in age-related conditions, given its involvement in regulating vascular tone, inflammation, oxidative stress, and tissue remodeling. In this context, repurposing widely used RAAS-modifying drugs for aging-related indications is an area of growing interest. Table 3 presents an overview of the major therapeutic classes and their mechanisms of action.

## Future directions and unanswered questions

RAAS, once seen mainly as a fluid and BP regulator, is now recognized as a key player in aging and age-related diseases. It intersects with several aging hallmarks, including inflammation and mitochondrial dysfunction, positioning it as a promising target for therapies aimed at extending lifespan and health span. However, despite advances in understanding RAAS in aging, critical gaps remain. Longitudinal studies are needed to define when and how RAAS shifts occur, and systemic biomarkers poorly reflect local tissue-specific activity, which remains underexplored due to limited measurement tools. The low specificity of antibodies for AT_1_R and AT_2_R limits accurate receptor profiling in experimental contexts (212). Additionally, the role of RAAS-targeting autoantibodies in aging, disease progression, and treatment resistance needs further investigation. Sex differences in RAAS activity also remain poorly characterized, despite hormonal influences that may affect disease risk and treatment outcomes. While RAASi are widely used in older adults, their effects on healthy aging, frailty, and cognition remain unclear. Furthermore, the development of antisenescent strategies brings unique clinical challenges. These include identifying optimal windows for intervention, selecting end points that meaningfully reflect functional aging outcomes, and determining appropriate trial durations in populations with heterogenous health trajectories. Clinical trial design must also consider comorbidities that accelerate biological aging and influence treatment responsiveness. Addressing these questions will be vital for the responsible translation of antisenescent medicines targeting RAAS and beyond. Future research should also focus on personalized approaches using biomarkers, autoantibody profiling, and genetic polymorphisms to optimize RAAS-targeted therapies from both the preventative and treatment perspectives in aging populations.

## Figures and Tables

**Table 3:**
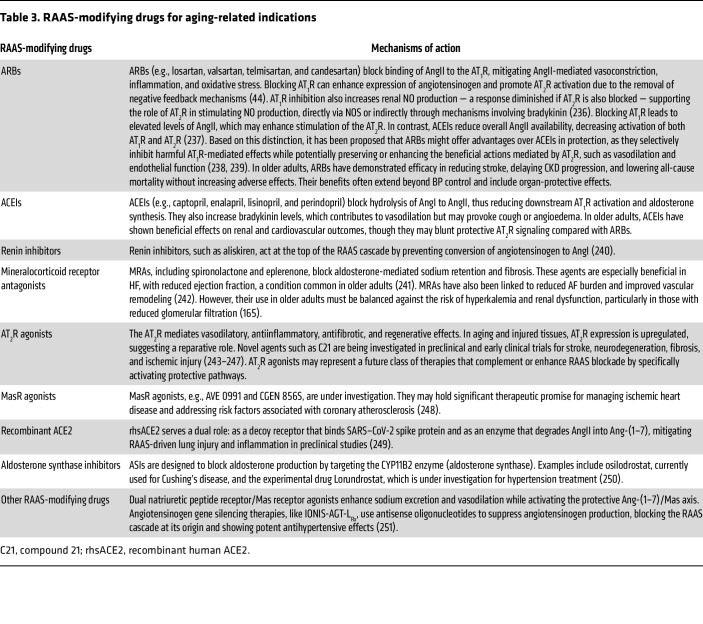
RAAS-modifying drugs for aging-related indications

**Table 2:**
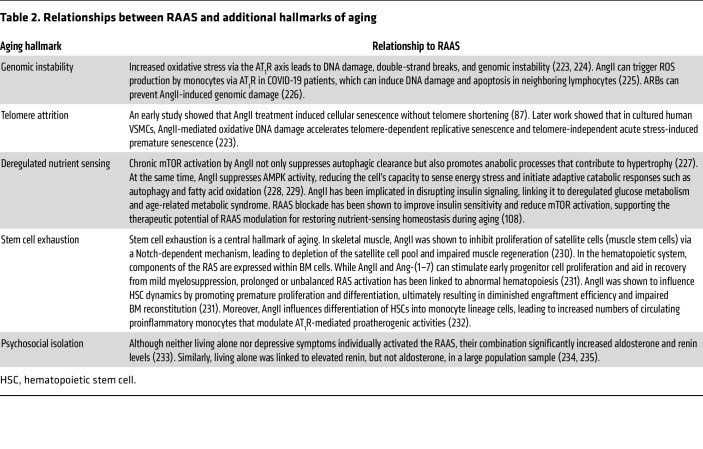
Relationships between RAAS and additional hallmarks of aging

**Table 1:**
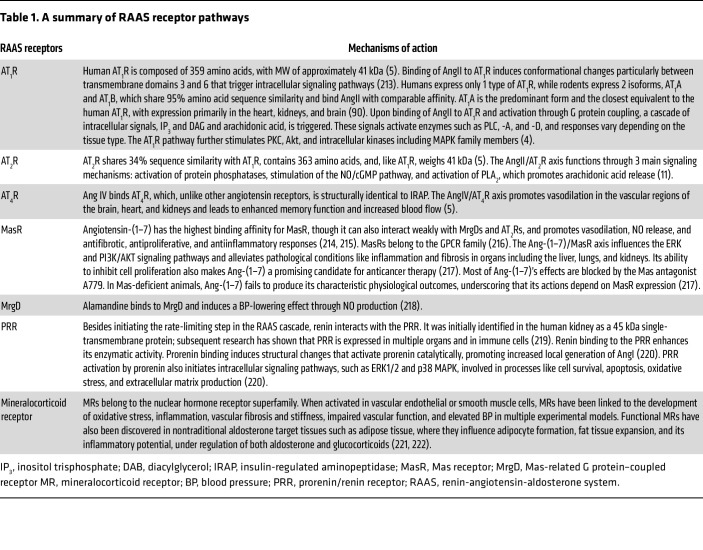
A summary of RAAS receptor pathways

## Version 1 11/03/2025

Electronic publication
